# Capillary morphogenesis gene 2 regulates adhesion and invasiveness of prostate cancer cells

**DOI:** 10.3892/ol.2014.2038

**Published:** 2014-04-04

**Authors:** LIN YE, ANDREW J. SANDERS, PING-HUI SUN, MALCOLM D. MASON, WEN G. JIANG

**Affiliations:** Metastasis and Angiogenesis Research Group, Institute of Cancer and Genetics, Cardiff University School of Medicine, Cardiff CF14 4XN, UK

**Keywords:** capillary morphogenesis gene 2, anthrax toxin receptor 2, prostate cancer, adhesion, invasion

## Abstract

Capillary morphogenesis gene 2 (CMG2), also known as anthrax toxin receptor 2, has been indicated in the formation of new vasculature and in the internalisation of the anthrax toxin. Anti-angiogenesis therapy that targets this molecule has been investigated. However, our recent studies of this molecule have indicated that this gene may also play certain roles in cancer cells. The present study aimed to examine the expression of CMG2 in prostate cancer tissues and cell lines, and also its impact on cellular functions. The expression of CMG2 was detectable in normal and prostate cancer tissues. The prostate cancer cell lines appeared to have relatively high expression compared with the prostatic epithelial cells. Knockdown of CMG2 impaired the adherence of the prostate cancer cells. CMG2 overexpression resulted in decreasing invasiveness, while the knockdown of CMG2 contrastingly enhanced this ability. The altered expression of CMG2 in the prostate cancer cells did not affect the *in vitro* or *in vivo* growth of the cells. Taken together, these results show that CMG2 is expressed in prostatic epithelia and cancer cells. In addition to its role in the angiogenesis and the internalisation of anthrax toxin, CMG2 also plays an important role in regulating the adhesion and invasion of prostate cancer cells.

## Introduction

Prostate cancer is the most common cancer in males in the UK and in the majority of Western countries, accounting for 40,975 cases of malignancies diagnosed in the UK and 10,721 mortalities, according to the cancer incidence and mortality rates of 2010 outlined by Cancer Research UK statistics (1). Although the majority of prostate cancers may remain indolent for years, 20–30% of affected patients are diagnosed with metastases in the UK. The distant metastases and local invasion remain major causes for the mortality and morbidity of the disease. Intensive research is being carried out to identify molecules and pathways for targeting cancer metastases. Adhesion molecules are among those molecules that play pivotal roles during the dissemination of cancerous cells (2). The transmembrane adhesion molecules, such as cluster of differentiation 44, and the intracellular adhesion associated molecules, such as focal adhesion kinase, have been indicated in the multiple steps of metastasis, particularly from prostate cancer (3,4). The present study aimed to examine capillary morphogenesis gene 2 (CMG2) in prostate cancer. This is a transmembrane protein that exhibits multiple functions, including adhesion and migration.

CMG2, also known as anthrax toxin receptor 2, has been identified as a gene that is upregulated in endothelial cells during tubule formation (5). Together with tumour endothelial marker-8 (TEM-8), this gene is a receptor of anthrax toxin that is able to mediate the internalisation of the toxin (6,7). Compared with TEM-8, which is selectively overexpressed during tumour angiogenesis, CMG2 is more widely expressed in normal tissues, including the heart, lung, liver, skeletal muscle, peripheral blood leukocytes, placenta, small intestine, kidney, colon and spleen (6). The discovery of TEM-8 and the importance of the anthrax toxin receptor, highlighted by the events of 9/11 in 2001, have fuelled the investigation of TEM-8 and CMG2 since the beginning of this century.

The CMG2 gene, located on chromosome 4q, encodes a protein of 489 amino acids with a putative signal peptide and extracellular, transmembrane and cytoplasmic domains (6). Mutations of the CMG2 gene have been identified in hyaline fibromatosis syndrome (HFS), including juvenile hyaline fibromatosis and infantile systemic hyalinosis, which are autosomal recessive syndromes characterized by multiple, recurring subcutaneous tumours, gingival hypertrophy, joint contractures, osteolysis and osteoporosis (8,9). Different natural variants encoded by alternatively spliced mRNA transcripts have been reported. The full-length protein product of this gene is CMG2^489^. In comparison to CMG2^489^, CMG2^488^ has 12 different amino acids at the cytoplasmic tail of the protein. CMG2^386^ lacks amino acids 213–233 of the full-length protein, and CMG2^322^ is predicted to be a secreted isoform of CMG2 lacking the transmembrane domain (6). The von Willebrand factor type A/inserted (vWA/I) domain with a metal ion-dependent adhesion site region in the extracellular section of the protein allows binding to the protective antigen (PA) subunit of the anthrax toxin to mediate the internalisation of the toxin. In addition to binding with PA, the extracellular domain also interacts with collagen IV, laminin and fibronectin (5).

CMG2 has been implicated in tumour-related angiogenesis (10) and to date, little is known about its role in cancer. The present study aimed to examine the expression of CMG2 in prostate cancer and the effect on cellular functions of prostate cancer cells.

## Materials and methods

PC-3 (European Collection of Animal Cell Culture, Salisbury, UK), DU-145, LNCapFGC, CA-HPV10 and PZ-HPV-7 (American Type Culture Collection, Mannasas, VA, USA), PNT1A and PNT2C2 (Professor Normal Maitland, University of York, York, UK) were routinely cultured in Dulbecco’s modified Eagle’s medium-F12 supplemented with 10% fetal calf serum and antibiotics (penicillin and streptomycin). Other reagents or kits were obtained from Sigma-Aldrich (Poole, UK).

Prostate tissue samples were available following collection from the patients at the Department of Urology, University Hospital of Wales (Cardiff, UK). The samples were snap-frozen in liquid nitrogen immediately after the surgery or biopsy. All protocols were reviewed and approved by the local ethical committee (Bro Taf Health Authority, Cardiff, UK) and all patients gave written informed consent.

### RNA extraction, reverse transcription polymerase chain reaction (RT-PCR) and quantitative PCR

Total RNA was isolated from frozen tissues and culture cells using TRI reagent (Sigma-Aldrich). RT-PCR was carried out using an RT kit with an anchored oligo (dT) primer supplied by AbGene (Thermo Scientific, Rockford, IL, USA), using 0.5 μg total RNA for each 20 μl RT reaction. The quality of cDNA was verified using GAPDH primers (sense, 5′-CAGGAGGTTGAAGGACTAAA-3′ and antisense, 5′-GGGATCAGTTTTCTTTGTCA-3′). Conventional PCR was performed with specific primers for CMG2 (sense, 5′-CAAAATCAGTAAAGGCTTGG-3′, and antisense, 5′-CAAAGGTTCTTCTTCCTCCT-3′). The conditions for the amplification were: 94°C for 5 min, followed by 35 cycles of 94°C for 30 sec, 55°C for 30 sec and 72°C for 1 min, and the final extension for 7 min at 72°C. The products were separated on 1.5% agarose gel followed by staining with ethidium bromide.

### Western blot analysis

The cells were lysed in a lysis buffer containing 1% Triton, 0.1% SDS, 2 mM CaCl_2_, 100 μg/ml phenylmethylsulfonyl fluoride, 1 μg/ml leupeptin and 1 μg/ml aprotinin for 60 min. Equal amounts of each protein sample (20 μg/lane) were separated in a 10% polyacrylamide gel. Following electrophoresis, the proteins were blotted onto nitrocellulose sheets. Subsequent to blocking in 10% skimmed milk for 60 min, the blots were probed with the polyclonal goat anti-human CMG2 (R&D Systems, Minneapolis, MN, USA) and peroxidase-conjugated secondary antibody. Protein bands were visualised using the Supersignal^TM^ West Dura system (Pierce Biotechnology, Inc., Rockford, IL, USA) and an UVITech imager (UVITech, Inc., Cambridge, UK).

### Altering the expression of CMG2 in prostate cancer cells

The mammalian CMG2 expression vectors were constructed using a pEF6/V5/His vector (Invitrogen, Paisley, UK) and used to transfect PC-3 cells by electroporation. Following transfection, the cells were selected using blasticidin (5 μg/ml). A subline named PC-3^CMG2exp^, which overexpressed CMG2, and a subline named PC-3^pEF^, which carried empty plasmid vectors, were also established. PC-3^WT^ was designated for the wild-type cells. Constructed vectors carrying anti-CMG2 ribozymes were cloned into the same plasmid vectors and used to knock down the expression of CMG2 in the PC-3 cells (PC-3^ΔCMG2^).

### In vitro cell growth assay

The cells were plated onto a 96-well plate (3,000 cells/well). Cell growth was assessed after one, three and five days. Crystal violet was used to stain the cells, and absorbance was determined at a wavelength of 540 nm using a spectrophotometer (Elx800; BioTek, Bedfordshire, UK).

### In vitro invasion assay

According to a standard procedure, Transwell inserts with an 8-μm pore size were coated with 50 μg Matrigel (BD Matrigel™ Basement Membrane Matrix; BD Bioscience, Oxford, UK) and air-dried. Following rehydration, 30,000 cells were added to each well. After 96 h, the cells that had migrated through the matrix to the other side of the insert were fixed, stained and then counted under a microscope.

### Cell-matrix adhesion assay

The cell-matrix adhesion assay was performed as previously described (3). In total, 40,000 cells were added to each well of the 96-well plate, which was pre-coated with Matrigel (5 μg/well). Subsequent to 40 min of incubation, the non-adherent cells were removed using a balanced salt solution buffer. The adhered cells were fixed, stained and then counted.

### Tumour growth in an athymic mouse model

Female athymic nude mice (4–8 weeks old; CD-1; Charles River Laboratories, Inc., Wilmington, MA, USA) were subcutaneously injected with PC-3 cells (1×10^6^) in Matrigel (2.5 mg/ml). The mice were kept in sterilised, filtered cages in 12-h dark/12-h light standardised environmental conditions approved by the local ethical committee. Tumours were measured twice a week using digital callipers and calculated as tumour volume = 0.512 × width^2^ × length (mm^3^).

### Statistical analysis

Two sample t-test was performed using the SPSS statistical software (version 18; SPSS, Inc., Chicago, IL, USA). P<0.05 was considered to indicate a statistically significant difference.

## Results

### Expression of CMG2 in prostate cancer tissues and cell lines

**Figure 1:**
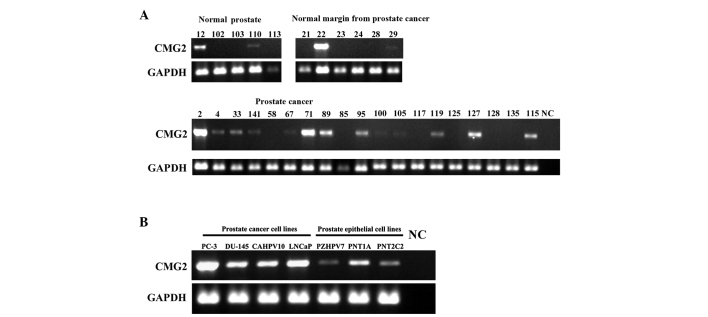
Expression of CMG2 in prostate tissues and cell lines. (A) CMG2 expression in human prostate tissues, examined using RT-PCR. (B) CMG2 is expressed by the prostate cancer cell lines. CMG2, capillary morphogenesis gene 2; RT-PCR, reverse transcription polymerase chain reaction; NC, negative control.

CMG2 has been detected in various cell types, including vascular endothelial cells, epithelial cells lining the lumen of the intestine and respiratory system and cells of the epidermis (5–7). In the present study, CMG2 mRNA was examined in the human prostate tumours and cell lines (Fig. 1). CMG2 transcripts were detected in two of five normal prostate tissues (40.0%), and also in two of the six background tissues of prostate cancer (33.3%). CMG2 appeared to be detectable in the majority of the prostate tumours (13/19, 68.4%). In line with the observations in the prostate tissues, CMG2 was expressed at relatively high levels in the four prostate cancer cell lines (PC-3, DU-145, CA-HPV-10 and LNCaP) compared with its expression in the three immortalised prostate epithelial cell lines (PZHPV-7, PNT1A and PNT2C2).

### Overexpression and knockdown of CMG2 in prostate cancer cells

**Figure 2:**
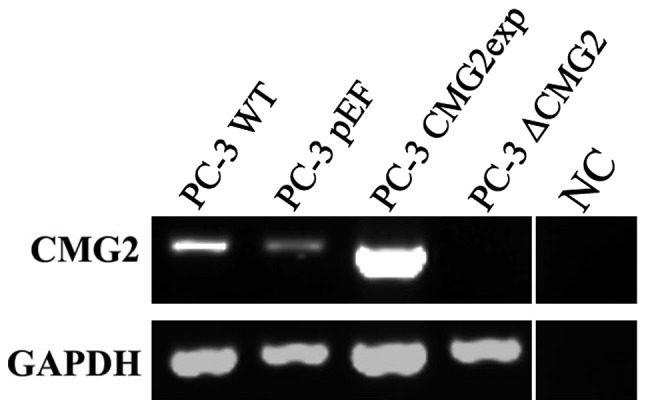
Altered expression of CMG2 in PC-3 cells was verified using RT-PCR. CMG2, capillary morphogenesis gene 2; RT-PCR, reverse transcription polymerase chain reaction; WT, wild-type; pEF, empty plasmid vector; NC, negative control; ΔCMG2, CMG2-knockdown; CMG2exp, CMG2 overexpression.

For assessing the effect of CMG2 on cellular functions, constructed plasmid vectors carrying either the full-length CMG2 coding sequence or anti-CMG2 ribozymes were transfected into the prostate cancer PC-3 cells. Following the selection with blasticidin, RNA was extracted and the CMG2 transcripts in the transfected cells were determined using RT-PCR. The overexpression and knockdown of CMG2 were verified in the respective transfectants (Fig. 2).

### Altered CMG2 expression and the effect on adhesion and invasion of prostate cancer cells

**Figure 3:**
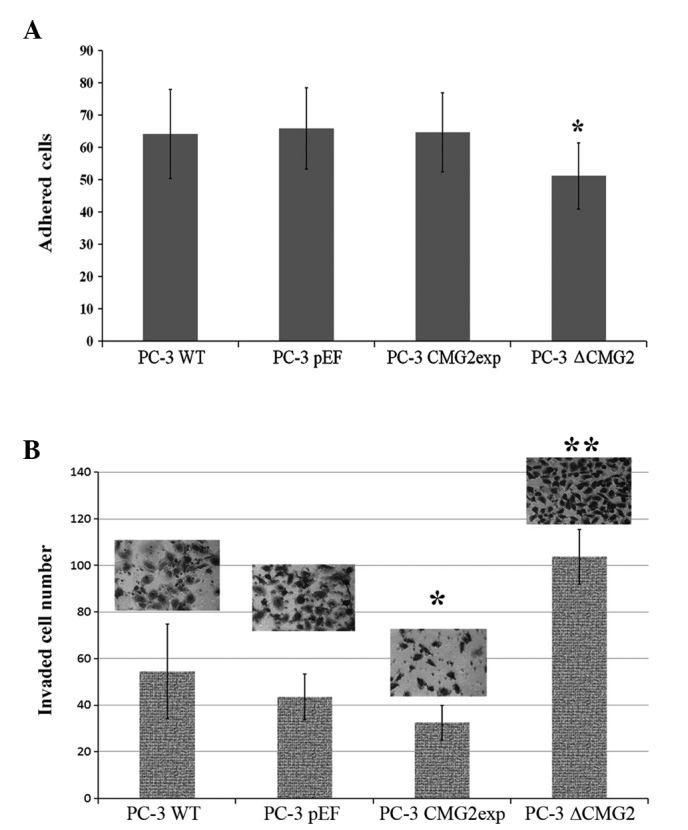
CMG2 affects the adhesion and invasion of prostate cancer cells. (A) Effect of CMG2 on adhesion. (B) CMG2 and invasiveness of prostate cancer cells. ^*^P<0.05 and ^**^P<0.01, vs. control. CMG2, capillary morphogenesis gene 2; WT, wild-type; pEF, empty plasmid vector; ΔCMG2, CMG2-knockdown; CMG2exp, CMG2 overexpression.

CMG2 has been shown to mediate the binding of prostate cancer cells to the extracellular matrix, mainly via interactions with laminin and collagen IV (5). In the present study, the adherence of prostate cancer cells to Matrigel was examined first (Fig. 3A). Overexpression of CMG2 did not enhance the adhesion of the prostate cancer cells to the Matrigel in comparison with the control cells, while the knockdown of CMG2 resulted in a significant decrease in matrix adherence.

Although reduced adhesion was observed in the CMG2-knockdown cells, a further examination of the invasiveness showed a significantly increased invasive capacity of the cells. In contrast to the increased invasion, an impaired invasive capacity was observed in the cells with overexpression of CMG2 (Fig. 3B).

### CMG2 and the growth of prostate cancer cells

**Figure 4:**
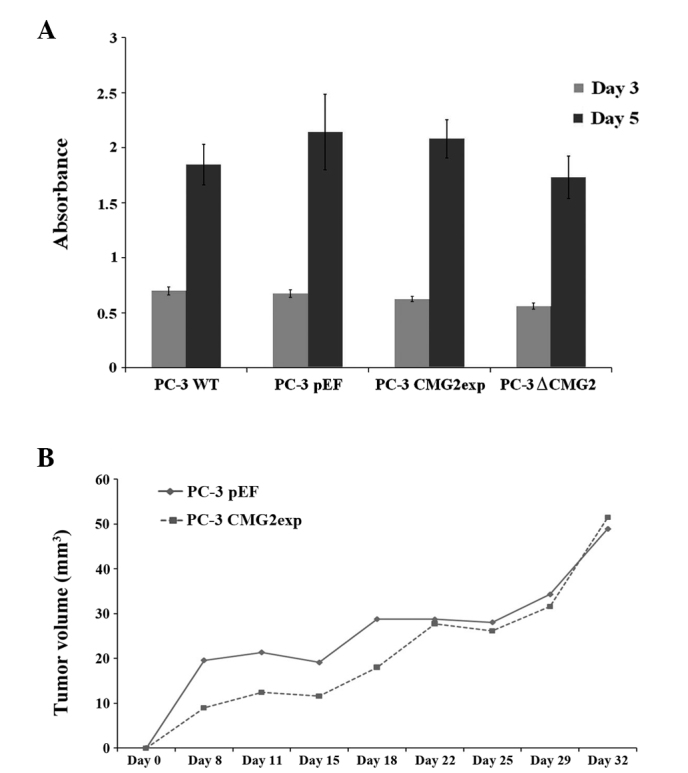
CMG2 and the growth of prostate cancer cells, (A) *in vitro* and (B) *in vivo*. CMG2, capillary morphogenesis gene 2; WT, wild-type; pEF, empty plasmid vector; ΔCMG2, CMG2-knockdown; CMG2exp, CMG2 overexpression.

The effect on cell growth was determined using an *in vitro* growth assay (Fig. 4A). No marked change was observed in the cells with altered CMG2 expression over the periods of three and five days. A further experiment using the mouse xenograft tumour model showed slower tumour growth of the cells with CMG2 overexpression at an earlier stage following the inoculation (Fig. 4B). The tumours grew to sizes similar to the control group over the 32-day period.

## Discussion

CMG2 has been identified as an upregulated gene in endothelial cells during angiogenesis, and as a receptor for the anthrax toxin (5,7). Mutations of this gene have been observed in HFS (9,11,12). Compared with the other receptor of the anthrax toxin, TEM-8, CMG2 exhibits a wider expression pattern that is observed in more tissue types (6). In contrast to the investigations of its roles in angiogenesis and *Bacillus anthracis* infection, little is known about the role of CMG2 in malignancies. Our recent study examined the expression of CMG2 in breast cancer and reduced expression was found to be associated with a shorter patient survival rate (unpublished data). In the present study, the expression of CMG2 in human prostate cancer tissues was first assessed using RT-PCR. The existence of the CMG2 transcripts was evident in the prostate cancer specimens, in which 68.4% of the samples appeared to be positive. CMG2 was found to be less frequently expressed in the normal prostate tissues (40.0%) and also the background tissues of prostate cancer (33.3%). To clarify the heterogeneity of CMG2 expression in the prostate tissues, the study further examined the expression of CMG2 in prostate cancer and prostatic epithelial cells using RT-PCR. CMG2 transcripts were highly expressed in the prostate cancer cell lines compared with the relatively low expression observed in the immortalised prostatic epithelial cell lines. This indicates that CMG2 may play a positive role in prostate cancer that may be different from its function in breast cancer.

Following the assessment of CMG2 expression in human prostate cancer tissues and cell lines, the study assessed the expression of CMG2 in one prostate cancer cell line, PC-3, which is one of the prostate cancer cell lines most commonly used in research. Due to the relatively low expression levels observed in the PC-3 cells in comparison with the other prostate cancer cell lines, the overexpression and knockdown of CMG2 were performed to provide double evidence for its functions in the prostate cancer cells. The extracellular section of CMG2 has a vWA domain, also called the I-domain, which is a well-characterised protein-protein interaction domain involved in cell adhesion that exists in extracellular matrix proteins and integrins (13). The homology shared in the vWA domain between CMG2 and integrins indicates a potential involvement of CMG2 in cell adhesion. An earlier study indicated specific possible binding proteins for CMG2 in the extracellular matrix, including collagen IV and laminin (5). In the present study, following the verification of the overexpression and knockdown of CMG2, the effect on cell adhesion was first determined. Reduced cell-matrix adhesion was observed in the cells with reduced CMG2 expression, and contrasting results were not observed in the cells with CMG2 overexpression. This indicates that CMG2 is involved in cell adhesion, but that above a certain threshold it makes no further contribution to the cell adhesion in the prostate cancer cells.

Mutations of CMG2 that are evident in HFS indicate that CMG2 plays a role in the maintenance of extracellular matrix homeostasis. A recent study has shown that CMG2 and TEM-8 can regulate the extracellular matrix via regulation of membrane type 1-matrix metalloproteinase (MMP) and MMP2 (14). All these results indicate a potential involvement of CMG2 in the invasion of prostate cancer cells. However, in the present study, it was observed that a different role may be played by CMG2 in the regulation of invasion of the prostate cancer cells. Following the overexpression of CMG2, inhibition of the invasion of the PC-3 cells was observed, while an enhanced level of invasion was observed in the CMG2-knockdown cells. This indicates that CMG2 may participate in the regulation of the invasion of prostate cancer cells via a different mechanism that is yet to be elucidated.

In addition to the effect on cell adhesion and invasion, the effects on cell growth were also examined. There was no obvious effect observed in the *in vitro* growth assays. For the *in vivo* tumour growth, a slower growth rate was observed at an earlier stage of tumour development following the subcutaneous inoculation for which the effect on cell invasion may account. However, the tumours all grew to a similar size at the end of the 32-day experimental period. This indicates that little impact was made by CMG2 on the *in vivo* tumour growth of prostate cancer cells.

In conclusion, CMG2 is expressed by prostate cancer cells and can regulate the adhesion and invasion of these cells. CMG2 may play a certain role during the dissemination of prostate cancer cells and has little impact on the *in vitro* and *in vivo* growth of these cells. However, the exact role played by CMG2 in prostate cancer and the possible relevance to sexual hormones are yet to be investigated in future studies.
